# Near-complete response to low-dose ceritinib in recurrent infantile inflammatory myofibroblastic tumour

**DOI:** 10.3332/ecancer.2021.1215

**Published:** 2021-04-01

**Authors:** Abhenil Mittal, Aarushi Gupta, Sameer Rastogi, Adarsh Barwad, Swati Sharma

**Affiliations:** 1 Department of Medical Oncology, Dr Bhim Rao Ambedkar (BRA) Institute Rotary Cancer Hospital, All India Institute of Medical Sciences, New Delhi 110029, India; 2 Department of Radiodiagnosis, ABVIMS and Dr RML Hospital, New Delhi 110001, India; 3 Department of Pathology, All India Institute of Medical Sciences, New Delhi 110029, India

**Keywords:** ceritinib, infant, inflammatory myofibroblastic tumour, low dose

## Abstract

**Background:**

Infantile inflammatory myofibroblastic tumour (IMT) is rare and the majority are driven by anaplastic lymphoma kinase (ALK) rearrangements. Previous literature on the use of ALK inhibitors in paediatric IMTs is extremely limited with no published literature on the use in infants. Crizotinib and ceritinib are two ALK inhibitors which are available and have been used in IMTs; however, ceritinib is much more affordable in the low- and middle-income country (LMIC) setting than crizotinib.

**Case:**

An 11-month-old child, who had undergone surgery for mesenteric IMT at the age of 3 months, had an unresectable recurrence with soft tissue deposits in the subdiaphragmatic location abutting the spleen and paravesical location. As surgery would have entailed splenectomy and partial cystectomy, she was treated with low-dose ceritinib (300 mg/m2/day) with which she had a near-complete response without any toxicity.

**Discussion and conclusion:**

This is the first report of the use of ceritinib at a lower dose for infantile IMT having immense practical applications for the low- and middle-income setting.

## Introduction

Inflammatory myofibroblastic tumours (IMTs) are considered to be potentially malignant tumours with a propensity for local recurrence and rarely metastasis [1, 2]. With increasing expertise in diagnosis, they have been described in almost all anatomical locations and in all age groups [3, 4]. Presentation in infancy is rare with description limited to small case series and case reports [3, 5–9]. Histologically, IMTs are characterised by a variably cellular spindle cell proliferation in a myxoid to collagenous stromal background with the predominance of inflammatory cells. Histology rarely correlates with prognosis, except for the rare aggressive epithelioid variant [3, 10]. Immunohistochemistry (IHC) is non-specific [3, 10]. The need for an experienced sarcoma pathologist for correct diagnosis cannot be overemphasised.

Surgery remains the treatment of choice for the localised disease [11, 12]. With the discovery of targetable alterations in the anaplastic lymphoma kinase (ALK) gene seen in around 50% of the cases of IMTs, precision oncology has come to the forefront of the management of IMTs. Crizotinib is now the standard of care in adult unresectable ALK-positive IMTs (defined as the presence of ALK rearrangement in >15% of tumour cells by dual probe break-apart fluorescence *in situ* hybridisation (FISH) or staining by IHC) [13]. However, literature on the use of ALK inhibitors in paediatric patients is limited. Only four infants have been treated with ALK inhibitors so far (all crizotinib), with three having a favourable response [10, 14]. However, crizotinib is expensive in India and is unaffordable for most patients, whereas generic ceritinib is easily available and much more affordable. Here, we report the first ever case of an infant girl child with recurrent ALK-positive IMT who had a near-complete response to low-dose ceritinib.

## Results

**Figure 1.**
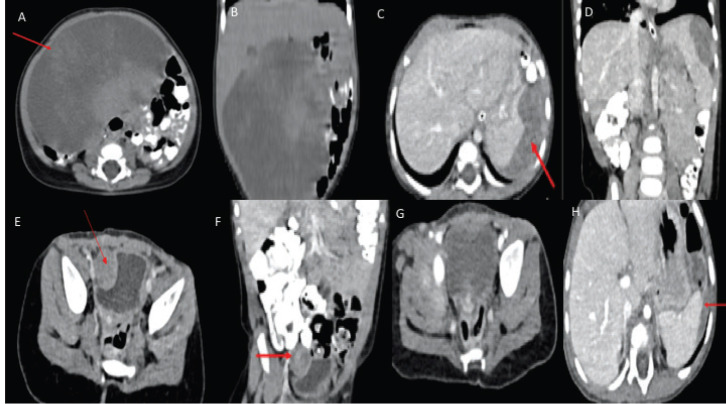
(a, b): Pre-operative CECT abdomen axial + coronal images showing a large hypodense mesenteric lesion with mild heterogeneous post-contrast enhancement displacing small bowel loops to the left side and ascending colon posteriorly and abutting inferior surface of liver with no obvious infiltration. (c, d): CECT abdomen at recurrence axial + coronal images showing a heterogeneously enhancing lesion in the left subdiaphragmatic region abutting the superior surface of the spleen with indentation and loss of fat plane. (e, f): CECT abdomen axial + coronal images showing a heterogeneously enhancing lesion in the right paravesical region indenting the right lateral wall of urinary bladder with loss of fat plane. (g, h): Two months post-Ceritinib CECT abdomen axial images showing complete resolution of a right paravesical lesion and near-complete resolution of the left subdiaphragmatic lesion.

**Figure 2.**
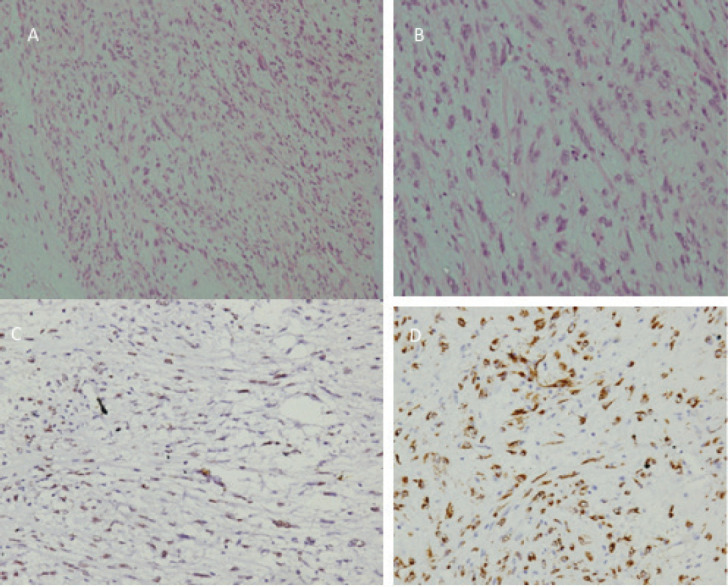
(a): Low-power photomicrograph of the tumour showing cells arranged in fascicles and a haphazard pattern with an oedematous background and admixed inflammatory cells. (b): High-power picture showing spindle cell population exhibiting myofibroblastic differentiation with mild-to-moderate nuclear pleomorphism, finely dispersed chromatin and moderate-to-abundant cytoplasm. The inflammatory cells are rich in plasma cells with lymphocytes and few oeosinophils (H&E 200×). (c): Immunostain for ALK-1 on D5F3 Ventana platform showing diffuse nuclear reactivity in 100% of the tumour cells with myofibroblastic differentiation. (d): Immunostain for SMA showing cytoplasmic reactivity in cells with myofibroblastic differentiation.

A 3-month-old female with an uncomplicated childbirth presented with a gradually progressive abdominal distension without any change in bowel habit or constitutional symptoms. Contrast-enhanced computed tomography (CECT) scan of chest, abdomen and pelvis showed a large ill-defined homogenous hypodense lesion of size 8.4 × 11.4 × 11.3 cm (APxTRAxSag), predominantly on the right side of the abdomen and in the midline showing mild heterogeneous post-contrast enhancement on delayed images (at 5 minutes) (Figure 1a and b). These findings were suggestive of a mesenteric mass, likely malignant. She underwent exploratory laparotomy with gross total excision of the mass and resection anastomosis of the involved small bowel. Histopathology showed a spindle cell tumour with cells arranged in a fascicular and haphazard pattern with abundant admixture of inflammatory cells rich in plasma cells, lymphocytes and few oeosinophils. The tumour cells showed mild-to-moderate pleomorphism with finely dispersed chromatin and moderate-to-abundant oeosinophilic cytoplasm. Variable mitosis was seen (4–5/10 per high-power field) (Figure 2a and b). Tumour cells showed diffuse nuclear immunoreactivity for ALK-1 protein (100%) on D5F3 Ventana platform and cytoplasmic positivity for smooth muscle actin (SMA) and desmin (Figure 2c and d). Hence, a diagnosis of infantile IMT was suggested. She developed abdominal pain 6 months after surgery and imaging (CECT) showed recurrent disease in right paravesical and left subdiaphragmatic regions (Figure 1e and f). As resection would have required debilitating surgery in the form of splenectomy and partial cystectomy, she was started on ceritinib 150 mg once a day (300 mg/m^2^) with food (the child was able to swallow the capsule), after discussion with the multidisciplinary tumour board. The child was monitored for toxicity with two weekly complete blood counts, liver and renal function tests for the first month, followed by monthly liver function testing. An electrocardiogram (ECG) was obtained prior to starting ceritinib, at 2 weeks of starting treatment and then monthly. Response assessment after 2 months showed a near-complete response with the disappearance of the paravesical lesion and 95% reduction of the subdiaphragmatic lesion (Figure 1g and h). A follow-up scan at 6 months of starting ceritinib showed complete response to therapy with no toxicity.

## Discussion

Prior to the discovery of the ALK gene rearrangements by Griffin *et al* [15] in 1999, the biology of IMTs was an enigma. Since then, studies in adult patients have showed that up to 50% of the patients harbour rearrangements in the ALK gene detected by either FISH or IHC, leading to constitutive activation of receptor tyrosine kinase and tumourigenesis [16]. Although EML4–ALK (echinoderm microtubule-associated protein-like 4–ALK) fusions are most commonly seen in non-small cell lung cancer (NSCLC), a set of unique translocations involving ALK has been described in IMT, including RANBP2–ALK (RAN binding protein 2–ALK) and CARS–ALK (cysteinyl t-RNA synthetase–ALK), which are not found in other tumours [17, 18]. With advances in molecular pathology, additional abnormalities have been characterised in IMTs, including ROS1, RET (rearrange during transfection) and NTRK (neurorophic tropomyosin receptor kinase) gene fusions [8, 10, 18]. In a recent case series of 62 cases of IMT, 35 patients (56%) were positive for ALK, 6 patients (10%) were positive for ROS1 and 1 patient had RET gene arrangement [19]. Out of these, ROS1 gene rearrangements were particularly more common in children (5/6 ROS1-positive cases were children). Majority of fusion-negative IMTs (90%) were adults indicating a high prevalence of driver fusions in paediatric IMTs.

Efficacy of crizotinib in ALK-positive IMTs was reported by Butrynski *et al* [20]. Concrete evidence of the efficacy of crizotinib was provided by the CREATE trial, in which 50% of the patients with ALK-positive IMTs responded to crizotinib with a median duration of response of 9 months and 73% of the patients were progression-free at 1 year [13]. However, efficacy in the paediatric population was not established as experience with ALK inhibitors was limited to NSCLC and adult IMTs.

The first relatively large study to report the efficacy of crizotinib in paediatric IMTs was published in 2017, in which a 36% response rate in 14 children with metastatic/unresectable IMT was found [21]. The recommended phase II dose of crizotinib was 280 mg/m^2^ (double that of adult dose) [22]. In a phase I study of ceritinib in 22 ALK-positive paediatric tumours (six IMTs) presented at American Society of Clinical Oncology (ASCO) 2015, in children more than 1 year of age, the recommended maximum tolerated dose was 510 mg/m^2^ without food. However, the median age in this study was 10 years (no infants were recruited) and toxicities were significant (86% all grade diarrhoea, 81% all grade vomiting, 54% elevated alanine transaminase and 40% decreased appetite) [23]. Brivio and Zwaan [24] demonstrated excellent responses to ceritinib in adolescent IMTs in their two cases; they used lower doses of 300 ﻿mg/m^2^ and 450 mg/m^2^. The manuscript did not mention whether ceritinib was given with food or empty stomach and although both patients responded, the one treated with 450 mg/m^2^ developed acute liver failure related to ceritinib [24]. This provided preliminary evidence of the efficacy of a lower dose of ceritinib in paediatric IMTs. Based on this evidence and previous data of lower dose ceritinib in NSCLC [25, 26], we treated our patient with 300 mg/m^2^/day ceritinib taken with low fat meal (150 mg/day) – similar to the adult dose of 450 mg/day. Had we used crizotinib at its recommended dose, the cost of therapy would have been around Rs. 40,000 ($550) per month, which would have been unaffordable for our patient. With this approach, we were able to cut down the cost by one-fifth to Rs. 8000/month ($110). She had improvement in symptoms within 2 weeks of starting therapy and a near-complete response in 2 months with no toxicity, thus showing promising efficacy of a lower dose of ceritinib. Based on previous published data in NSCLC and our experience, we believe that low-dose ceritinib is an excellent cost-effective alternative to crizotinib which needs to be explored further in larger studies, especially in an LMIC setting.

## Conclusion

With advancements in molecular pathology, precision oncology is the future to managing this very rare tumour. Published data describe the efficacy of ALK inhibitors predominantly in adults. Recommended doses in children seem higher than required for optimal response with unwanted toxicity and cost. This is the first case to describe complete response to ceritinib in infantile IMTs at a lower than recommended dose and has immense practical implications, especially for LMICs.

## Abbreviations

IMT, Inflammatory myofibroblastic tumour; ALK, Anaplastic lymphoma kinase; IHC, Immunohistochemistry; SMA, Smooth muscle actin; FISH, Fluorescence *in situ* hybridisation; NSCLC, Non-small cell lung cancer; LMIC, Low- and middle-income country

## Consent

Taken from parents.

## Conflicts of interest

None.
